# Skeletal Dysplasias: Growing Therapy for Growing Bones

**DOI:** 10.3389/fphar.2017.00079

**Published:** 2017-03-06

**Authors:** Angie C. Jelin, Elizabeth O'Hare, Karin Blakemore, Eric B. Jelin, David Valle, Julie Hoover-Fong

**Affiliations:** ^1^Gynecology and Obstetrics, Johns Hopkins University School of MedicineBaltimore, MD, USA; ^2^Johns Hopkins UniversityBaltimore, MD, USA; ^3^Pediatric Surgery, Johns Hopkins University School of MedicineBaltimore, MD, USA; ^4^Genetics, Johns Hopkins University School of MedicineBaltimore, MD, USA

**Keywords:** hypophosphatasia, mucopolysaccharidosis, osteogenesis imperfecta, achondroplasia, *Asfotase alfa*, enzyme replacement therapy

## Abstract

Skeletal dysplasias represent a large and diverse group of rare conditions affecting collagen and bone. They can be clinically classified based on radiographic and physical features, and many can be further defined at a molecular level (Bonafe et al., 2015). Early diagnosis is critical to proper medical management including pharmacologic treatment when available. Patients with severe skeletal dysplasias often have small chests with respiratory insufficiency or airway obstruction and require immediate intubation after birth. Thereafter a variety of orthopedic, neurosurgical, pulmonary, otolaryngology interventions may be needed. In terms of definitive treatment for skeletal dysplasias, there are few pharmacotherapeutic options available for the majority of these conditions. We sought to describe therapies that are currently available or under investigation for skeletal dysplasias.

## Introduction

A skeletal dysplasia is diagnosed in 1 of 5,000 births each year (Orioli et al., 1986). Severity is highly variable and can range from mild short stature to perinatal lethality. Among patients with less severe forms that allow survival are many who will require significant care for the duration of their lives. Despite research in the area of skeletal dysplasias that has elucidated a great deal about the genetic pathophysiology of these conditions, there remain limited therapeutic options.

The ability to accurately diagnose a skeletal dysplasia *in utero* has improved in recent years due to advances in prenatal ultrasound and molecular diagnosis. There are, however, limitations to ultrasound; certain skeletal dysplasias may not be seen until the third trimester (e.g., some forms of osteogenesis imperfecta), while others may manifest as late as infancy or early childhood (e.g., pseudoachondroplasia). In general, the earlier the dysplasia manifests, the more significant are its effects in terms of prenatal and postnatal morbidity and mortality. When sonographic findings are present, parents benefit from prenatal counseling and anticipatory guidance. Understanding the inheritance pattern is also important to direct recurrence risk estimates for future pregnancies, particularly when both parents are affected with a skeletal dysplasia. Inheritance depends on the specific dysplasia and may include autosomal dominant, autosomal recessive and X-linked inheritance, or a combination of these when both parents are affected.

Medical management of patients with skeletal dysplasias is dictated by the underlying pathogenesis along with the type and severity of physical manifestations in the realm of orthopedics, neurosurgery, otolaryngology, and pulmonary services. Pharmacologic treatment for these conditions is disease-specific (Table 1), and 4 conditions/groups will be discussed.

**Table 1:** **Therapeutic options for skeletal dysplasias**.

	**Clinical therapy**	**Investigational/other therapy**
Osteogenesis Imperfecta	Bisphosphonates, growth hormone, Teriparatide	*In utero* stem cell transplantation, Raloxifene, Denosumab, Sclerastin, Anti-TGFβ
Hypophosphatasia	*Asfotase alfa*	Human parathyroid hormone^*^, bone marrow transplant^*^, gene therapy^*^
Mucopolysaccharidosis (MPS)		Stem cell transplantation, chaperone therapy, gene therapy
MPS Type I	Laronidase	Bone marrow transplant
MPS Type II	Idursulfase	
MPS Type IVa	*Elosulfase alfa*	
MPS Type VI	*Galsulfase*	
Achondroplasia		C-type natriuretic peptides, meclozine, human parathyroid hormone, human growth hormone

* *No longer employed, or under investigation*.

## Osteogenesis imperfecta

Osteogenesis imperfecta (OI), also known as “brittle bone disease,” is characterized by short stature, bone deformation, bone fragility, and osteoporosis (Forlino et al., 2011). There are over a dozen types of OI with a prevalence of 1–2 per 20,000 children (Yap and Savarirayan, 2016). Pathogenic mutations in OI have been uncovered in the following genes: *COL1A1, COL1A2, SERPINF1, CRTAP, LEPRE1, PPIB, SERPINHI*, or *FKBP10*. The majority of cases are due to an autosomal dominant mutation in *COL1A1* and *COL1A2* resulting in types I–IV, classified based on severity (Yap and Savarirayan, 2016). These mutations affect formation of type I collagen, which is the most prominent connective tissue in bone and skin.

Type I patients have a quantitative deficiency of collagen and often have blue sclerae and fractures without deformity. Patients with Types II–IV have defective forms of collagen caused by improper formation of the triple helix resulting in continual remodeling with a dominant negative effect. Type II OI is perinatally lethal, due to severe intrauterine fractures and bone deformities (Harrington et al., 2014). Type III is the most severe nonlethal type of OI, presenting with normal mental capacity (Krakow, 2015) and progressive deformities resulting in loss of ambulatory abilities (Krakow, 2015). Type IV OI is milder than type III, but still severe, commonly characterized by vertebral fractures and short stature (Krakow, 2015). Types II and III are generally detectable *in utero* (Krakow et al., 2008; Krakow, 2015), whereas the milder types of OI, types I, IV, and V, are usually diagnosed after birth (Harrington et al., 2014). Patients with type II and type III OI likely have a small chest cavity, and may require mechanical ventilation if demise is not inevitable secondary to severe fractures (Harrington et al., 2014).

In addition to the autosomal dominant mutations in COL1A1 and COL1A2, 5–10% of cases are due to a mutation in another gene. Type V OI, due to an autosomal dominant mutation in IFTITM5, is recognized by hypercallus formation and calcification of the intraosseous membrane (Liu et al., 2016). An autosomal recessive mutation in SERPINF1 is responsible for type VI (Harrington et al., 2014). Additional types of OI are autosomal recessive and are not well-recognized because they are very rare. Examples include the following types and (genes): type VII (CRTAP), type VIII (LEPRE1), type IX (PPIB), type X (SERPINH1), and Type XI (FKBP10; Harrington et al., 2014; Krakow, 2015).

Of the skeletal dysplasias, osteogenesis Imperfecta currently has the most pharmacologic treatment options. Overall the goal is to improve bone density and decrease fractures, and there are several drug families currently employed to do this (Lee et al., 2016). Medications include bisphosphonates, growth hormones, denosumab, and teriparatide. Additional therapies are under investigation; Raloxifine improves outcomes in murine models, and mesenchymal bone marrow transplant has promise when initiated *in utero*.

### Bisphosphonates

Bisphosphonates are one of the major pharmacotherapeutic agents clinically prescribed for OI (Dwan et al., 2014; Harrington et al., 2014). Bisphosphonates suppress bone remodeling and inhibit calcification by inactivating osteoclasts (Dwan et al., 2014; Harrington et al., 2014), which, in turn, decreases areal bone mineral density (aBMD), primarily in the spine, hip, and femur and decreases the incidence of fractures (Ward et al., 2010). There is variation in fracture incidence, aBMD, and bone pain among these agents (alendronate, pamidronate, etc.; Dwan et al., 2014) and in their route of administration (intravenous vs. oral). Adverse side effects to long-term use of bisphosphonates have been found, including cumulative micro damage, cartilage calcification (Sinder et al., 2013; Vasanwala et al., 2016), and osteonecrosis of the jaw in elderly patients (Dwan et al., 2014; Harrington et al., 2014). Bisphosphonates have been found to be less effective after 2–4 years of treatment, so intermittent treatment may be more beneficial in some cases.

### Growth hormone

Osteogenesis imperfecta is not typically associated with growth hormone deficiency, but growth hormone treatment can be beneficial by increasing aBMD and growth velocity in children with OI (Antoniazzi et al., 2010). In a randomized study, recombinant growth hormone (rGH) was combined with bisphosphonate therapy (Antoniazzi et al., 2010). Although, the rate of fractures did not differ, lumbar spine and wrist bone mineral density increased. There was no apparent decrease in fracture rate overall (Harrington et al., 2014), yet the combination of rGH and bisphosphonates has not been shown to increase fracture rate either (Antoniazzi et al., 2010).

### Teriparatide

Teriparatide is a bone stimulating recombinant form of parathyroid hormone used in anabolic therapy to treat osteoporosis (Vahle et al., 2002; Orwoll et al., 2014). When given in concert with bisphosphonate therapy, teriparatide has been shown to increase aBMD (Orwoll et al., 2014) and accelerate the healing of fractures (Rozen et al., 2007) in adults with Type I OI. In more severe forms of OI (types III and IV), teriparatide treatment showed no increase in aBMD compared to control groups (Orwoll et al., 2014).

### Future OI therapies

Development of OI treatments is ongoing. Studies have demonstrated an increase in bone healing by callus formation when teriparatide is combined with BMP-7, a recombinant protein in bones (Morgan et al., 2008). *Raloxifene* has been shown to decrease the rate of bone fractures in mice (Berman et al., 2016) and may prove to be useful to decrease fractures in future human trials of OI. *Denosumab* is an antibody currently used to prevent fractures in post-menopausal women with osteoporosis (Cummings et al., 2009; Shaker et al., 2015) and is being evaluated to treat OI. *Sclerostin antibody* has been shown in studies to increase bone formation, therefore improving bone mass (Sinder et al., 2013; Shaker et al., 2015), which is essential to OI preventative measures. *Anti-TGF*β *therapy* may also prove to be beneficial in decreasing osteoblast signaling and bone resorption (Shaker et al., 2015) in future OI treatment.

There are presently no clinically available *in utero* therapies for any of the skeletal dysplasias, however *bone marrow* and *mesenchymal stem cell transplantation* are currently under study for *in utero* treatment of OI (Mehrotra et al., 2010; Harrington et al., 2014). Case series demonstrate safety, with transient improvements in bone growth and decreases in fractures (Chan and Götherström, 2014). A larger clinical trial is underway (Chitty et al., 2016).

## Hypophosphatasia

Hypophosphatasia (HPP) is a rare metabolic disorder resulting from a loss-of-function mutation in the ALPL gene with corresponding deficiency of tissue-nonspecific alkaline phosphatase (TNSALP) (Millán and Plotkin, 2012; Whyte et al., 2015; Yap and Savarirayan, 2016). There are 6 recognized clinical forms of HPP with varied severity, all correlated to insufficient mineralization of bone and teeth as well as osteomalacia in adults (Yap and Savarirayan, 2016). Although, mild forms of HPP are found in adolescents and adults, HPP that manifests in the fetus is almost always associated with infantile and perinatal lethality (Millán and Plotkin, 2012; Yap and Savarirayan, 2016) due to abnormal skeletal development and respiratory complications resulting from a small chest with pulmonary hypoplasia (Nishioka et al., 2006).

### Current treatments for HPP

*Asfotase alfa* is a human recombinant TSNALP currently used to safely treat HPP by reestablishing TSNALP levels for proper degradation of inorganic pyrophosphate and consequential regulated bone mineralization (Nishioka et al., 2006; Whyte et al., 2016; Yap and Savarirayan, 2016). One study of this subcutaneous form of enzyme replacement therapy has demonstrated increased strength and agility as a result of improved bone mineralization (Whyte et al., 2016). An immense increase in perinatal and postnatal survival rates has been observed in patients treated with *asfotase alfa* (Whyte et al., 2016). In an open label study, infants with a previously perinatal lethal condition who were treated with *asfotase alfa* survived to have average stature with mainly defects in tooth enamel (Whyte et al., 2012).

### Potential treatments for HPP

Prior to the availability of *asfotase alfa*, several treatments were under investigation for HPP. These included *bone marrow transplants* (Millán and Plotkin, 2012), *parathyroid hormone (PTH)* treatment (Millán and Plotkin, 2012), and *fetal gene therapy*. Although, fetal gene therapy appeared to improve postnatal development in murine models (Sugano et al., 2011), its potential benefits are no longer under investigation due to the dramatic clinical benefits of enzyme replacement therapy with *asfotase alfa*.

## Mucopolysaccharidosis

Mucopolysaccharidoses (MPSs) are a type of lysosomal storage disease, a rare group of disorders that results in symptoms secondary to a defect in lysosomal function leading to abnormal storage of glycosaminoglycans (GAGs) in the bones, heart, brain, liver, or spleen (Muenzer, 2014; Regier and Tanpaiboon, 2016). The enzyme deficiency is specific to the type of MPS. MPSs are progressive, so most cases become lethal as the patient ages (Walkley, 2009) as GAG builds up and causes multiple organ failures (Muenzer, 2011). GAGs are also involved in complex secondary signaling pathways which can create permanent cellular damage (Muenzer, 2014), so early diagnosis and treatment are essential to patient longevity (Clarke, 2011). Early diagnosis, however, is often difficult in patients with normal cognitive abilities (Muenzer, 2011; Lachman et al., 2014; Regier and Tanpaiboon, 2016). There are seven types of MPS, each of which is caused by an autosomal recessive disorder, except for MPS II, which is X-linked recessive and generally occurs only in males (Valayannopoulos and Wijbug, 2011; Muenzer, 2014). Current treatments for the mucopolysaccharidoses are focused mostly on enzyme replacement therapy (ERT) and hematopoietic stem cell transplantation (HSCT; Clarke, 2011).

*Enzyme replacement therapy (ERT)* involves intravenous administration of the deficient enzyme specific to each type of MPS (Valayannopoulos and Wijbug, 2011; Haneef and Doss, 2016). ERT is currently used to treat MPS I, MPS II, and MPS VI (Muenzer, 2014; Haneef and Doss, 2016). Its use in the treatment of MPS IV type A (Morquio) is under *observational* study now that it has been FDA approved (Haneef and Doss, 2016; Regier and Tanpaiboon, 2016). Although, repeated administration ERT may improve some of the symptoms of MPS including respiratory function and mobility, it cannot reverse existing skeletal disease (Muenzer, 2014).

*MPS I* is caused by deficiency of α-L-iduronidase (IDUA; Wang et al., 2009; Valayannopoulos and Wijbug, 2011; Ou et al., 2016), a critical enzyme in the GAG degradation pathway of heparin sulfate and dermatan sulfate (Kakkis et al., 2001; Muenzer, 2011; Regier and Tanpaiboon, 2016). MPS I is categorized into 3 groups based on its presentation and severity: Hurler's syndrome (severe; lethal by age 10); Hurler-Scheie syndrome (moderate; lethality by age 20); and Scheie's syndrome (mild; possible normal life span; Kakkis et al., 2001). Larodinase is used in ERT for MPS I, and has been shown to safely reverse some MPS symptoms, not including those relating to skeletal disease (Wraith et al., 2004; Muenzer, 2014).

*MPS II* is the only form of MPS that is inherited as an X-linked recessive disorder. It results from a deficiency of iduronate-2-sulphatase (Muenzer, 2011) which, similar to MPS I, leads to insufficient degradation of dermatin sulfate and heparin sulfate (Muenzer, 2011; Haneef and Doss, 2016; Motas et al., 2016). Idursulfase administration has resulted in reduced non-central nervous system symptoms of MPS II (Haneef and Doss, 2016).

MPS *IVA* and *VI* are difficult to distinguish because of their varied presentation (Lachman et al., 2014), however treatment is dependent on the diagnosis. MPS IVA results from deficiency of N-acetylgalactosamine-6-sulfatase (GALNS) corresponding to the buildup of keratan sulfate (KS) and chondroitin-6-sulfate (C6S) (Regier and Tanpaiboon, 2016). Clinical treatment for MPS IVA with *elsosulfase alfa* is underway (Regier and Tanpaiboon, 2016). *Galsulfase* is currently the only treatment for *MPS VI* and has been successful in improving pulmonary function and mobility (Motas et al., 2016).

*Hematopoietic stem cell transplantation (HSCT)* and *bone marrow transplantation* are used to introduce unaffected donor cells into the body and correct the lysosomal disorder by the accompanied addition of the enzyme that was previously lacking (Haneef and Doss, 2016). HCST is recommended in treating Hurler syndrome (MPS I) with ERT pre-treatment for the best results (Clarke, 2011; Muenzer, 2014). HSCT has been less successful in patients with MPS II or severe forms of MPS III (Muenzer, 2014; Motas et al., 2016). Bone marrow transplant was effective in reversing CNS symptoms (Walkley et al., 1994) in MPS I, MPS II, MPS VI, and MPS VII but is associated with a high complication rate (Haneef and Doss, 2016).

### Future therapies for MPS

*Chaperone therapy* could potentially treat MPS by assisting in the proper folding of proteins to create functional enzymes that break down GAG (Kakkis et al., 2001). *Gene therapy* is promising in finding a cure for skeletal dysplasias if the mutated gene can be correctly identified and modified to produce functional proteins, but development of this technique will take continued research (Kakkis et al., 2001).

## Achondroplasia

Achondroplasia (ACH) is an autosomal dominant disorder generally resulting from a specific gain-of-function mutation (G380R) in the fibroblast growth factor receptor 3 (*FGFR3*) (Wang et al., 2013; Krakow, 2015; Yap and Savarirayan, 2016). As the most common type of nonlethal skeletal dysplasia, occurring in 1–2 of every 20,000 live births (Savarirayan and Rimoin, 2002; Yasoda et al., 2009; Klag and Horton, 2016), ACH is the cause for most cases of dwarfism (Klag and Horton, 2016; Yap and Savarirayan, 2016). The common clinical presentation of ACH includes short stature, relative macrocephaly with frontal bossing (Matsushita et al., 2014), and rhizomelic limb shortening (Faruqi et al., 2014; Krakow, 2015). There are usually no cognitive impairments associated with ACH unless symptoms result due to a complication of another manifestation (Gordon, 2000). Severe symptoms may result due to spinal stenosis at the foramen magnum.

Abnormal long-bone development typically is not obvious until after the first half of the second trimester thus, early prenatal diagnosis of ACH by ultrasound is not common (Krakow et al., 2008; Krakow, 2015). When achondroplasia is suspected, prenatal molecular diagnosis can be employed to evaluate for the specific G380R mutation, however sequencing of *FGFR3* may be preferred if hypochodroplasia is on the list of differential diagnoses. Affected parents may also desire prenatal molecular diagnosis or pre-implantation genetic diagnosis to evaluate for a known maternal or paternal mutation (Wang et al., 2013).

Pharmacologic treatments for ACH are currently in stage 2 clinical trials (Wendt et al., 2015; Klag and Horton, 2016). Treatments are aimed at regulating the function of FGFR3 in growth plate formation (Matsushita et al., 2014). FGFR3 negatively regulates bone growth (Faruqi et al., 2014). Its elevated function leads to irregular endochondral ossification (Wendt et al., 2015; Yap and Savarirayan, 2016) and underdeveloped linear bone growth (Liu et al., 2016) resulting from interrupted differentiation of chondrocytes (Yasoda et al., 2009; Wendt et al., 2015; Klag and Horton, 2016).

*C-type natriuretic peptides (CNP)* increased linear bone growth in murine models with ACH by antagonizing FGFR3 signals (Wang et al., 2013; Faruqi et al., 2014; Klag and Horton, 2016). Most CNPs are active in the body for less than 3 min (Klag and Horton, 2016) before they are removed by the natriuretic clearance receptor (NPR C) and neutral endopeptidase (NEP) (Wendt et al., 2015). Constant intravenous infusions of these short-lived CNPs would be required to observe any improvement of endochondral ossification in patients (Yasoda et al., 2009; Wendt et al., 2015). *BMN 111* (vosoritide) is a type of CNP that is resistant to digestion by NEP, and therefore has a longer circulation period in the body (Klag and Horton, 2016). Recent studies have shown an increased annual linear bone growth in cynomolgus monkeys (Wendt et al., 2015) and in children with ACH after daily subcutaneous administration of BMN 111 (Klag and Horton, 2016). BMN 111 is the most promising of CNP treatments and the only treatment for ACH that has made it to clinical trials (Klag and Horton, 2016).

### Possible future treatments of achondroplasia

*Meclozine* is an oral antihistamine that has been shown to block negative signaling of FGRF3 in chondrocytes (Matsushita et al., 2014; Klag and Horton, 2016) and increase linear bone growth in mice, both with ACH and wild-type (Matsushita et al., 2014). Furthermore, intermittent injection of *human parathyroid hormone [PTH]* in mice was accompanied by increased chondrogenesis and recovered bone growth (Xie et al., 2012). *Human growth hormone (hGH)* can increase short-term bone growth velocity in children, but is an ineffective therapy in adults (Savarirayan and Rimoin, 2002; Yasoda et al., 2009; Matsushita et al., 2014). A short-term study found increased chondrogenesis by *statin* administration, specifically rosuvastatin (Yamashita et al., 2014), but long-term analysis has not been done.

## Conclusion

The currently available clinical therapies for patients with skeletal dysplasia are predominantly palliative in nature, however enzyme replacement therapy is now available for certain skeletal conditions. The ability to perform enzyme replacement therapy requires knowledge of the underlying molecular diagnosis as well as the pathogenic pathway by which the mutation affects bone growth and/or development. In addition to enzyme replacement therapy, bone marrow transplant is a less specific form of therapy that is clinically beneficial for several skeletal dysplasias. It is also being trialed *in utero* for osteogenesis imperfecta. A lack of suitable biomarkers accounts for the deficiency of therapeutic treatments (Briggs et al., 2015). Identifying these biomarkers will enable more useful treatment methods, but there is also associated difficulty with targeting the specific mutations, even when their identity is known (Tomatsu et al., 2013). Advances in molecular technology enable a more rapidly confirmed diagnosis as well as an earlier diagnosis in childhood and even *in utero*. This is key to allow for useful therapies specific to the underlying diagnosis. Our diagnostic capabilities will allow for a more personalized approach to treatment and targeted gene therapy as a foreseeable approach in the future.

## Author contributions

All authors contributed to the concept and design of this project. All authors have reviewed and edited the final manuscript.

## Funding

AJ is funded by the Johns Hopkins Women's Health Scholar Program.

### Conflict of interest statement

The authors declare that the research was conducted in the absence of any commercial or financial relationships that could be construed as a potential conflict of interest. The reviewer SKJ and handling Editor declared their shared affiliation, and the handling Editor states that the process nevertheless met the standards of a fair and objective review.
